# National doping prevention guidelines: Intent, efficacy and lessons learned - A 4-year evaluation

**DOI:** 10.1186/s13011-016-0079-9

**Published:** 2016-10-10

**Authors:** Pia-Maria Wippert, Michael Fließer

**Affiliations:** Sociology of Health and Physical Activity, Department of Health Science, University of Potsdam, Am Neuen Palais 10, House 12, 14469 Potsdam, Germany

**Keywords:** Doping, Anti-doping program, Anti-doping guideline, Elite sports schools

## Abstract

**Background:**

Doping presents a potential health risk for young athletes. Prevention programs are intended to prevent doping by educating athletes about banned substances. However, such programs have their limitations in practice. This led Germany to introduce the National Doping Prevention Plan (NDPP), in hopes of ameliorating the situation among young elite athletes. Two studies examined 1) the degree to which the NDPP led to improved prevention efforts in elite sport schools, and 2) the extent to which newly developed prevention activities of the national anti-doping agency (NADA) based on the NDPP have improved knowledge among young athletes within elite sports schools.

**Methods:**

The first objective was investigated in a longitudinal study (Study I: t0 = baseline, t1 = follow-up 4 years after NDPP introduction) with *N* = 22 teachers engaged in doping prevention in elite sports schools. The second objective was evaluated in a cross-sectional comparison study (Study II) in *N* = 213 elite sports school students (54.5 % male, 45.5 % female, age *M* = 16.7 ± 1.3 years (all students had received the improved NDDP measure in school; one student group had received additionally NADA anti-doping activities and a control group did not). Descriptive statistics were calculated, followed by McNemar tests, Wilcoxon tests and Analysis of Covariance (ANCOVA).

**Results:**

Results indicate that 4 years after the introduction of the NDPP there have been limited structural changes with regard to the frequency, type, and scope of doping prevention in elite sport schools. On the other hand, in study II, elite sport school students who received further NADA anti-doping activities performed better on an anti-doping knowledge test than students who did not take part (*F(1, 207) =* 33.99, *p* <0.001), although this difference was small.

**Conclusion:**

The integration of doping-prevention in elite sport schools as part of the NDPP was only partially successful. The results of the evaluation indicate that the introduction of the NDPP has contributed more to a change in the content of doping prevention activities than to a structural transformation in anti-doping education in elite sport schools. Moreover, while students who did receive additional education in the form of the NDPP“booster sessions” had significantly more knowledge about doping than students who did not receive such education, this difference was only small and may not translate to actual behavior.

**Electronic supplementary material:**

The online version of this article (doi:10.1186/s13011-016-0079-9) contains supplementary material, which is available to authorized users.

## Introduction

The use of doping by young athletes is a concern in anti-doping work [1, 2]. In Germany, for example, the prevalence rate of doping among young amateur athletes is estimated to be 15 % [3], although actual rates may be higher as social desirability concerns may lead to underreporting of actual use. It has been estimated that within elite-sport schools in Germany, the earliest age for anabolic steroid and amphetamine use is between 11 [4] and 12 years [5]. Because adolescents develop their moral comprehension before the onset of puberty, i.e., before the age of 16 [6], it has been suggested that to enhance its impact, doping prevention should be timed to coincide with their moral development. For this reason, several anti-doping programs specifically targeting young people have been developed. For example, the gender-specific U.S. college anti-doping programs ATLAS and ATHENA [7–9] targeted the basic risk factors for doping use (e.g., knowledge, intention, attitudes/beliefs, individual factors, body image and skills) and contained instructional units on topics such as nutrition, alternatives to doping, and role-playing games in a college setting. The evaluation of these programs indicated that the program was successful in reducing interest in doping substances, decreased the probability of (self-reported) use, and led to a higher awareness of alternatives to doping [10]. However, a recent meta-analysis of existing randomized controlled trials (RCT’s) of ATLAS and ATHENA indicated that while such programs are effective in reducing doping intentions, they had less impact on actual behavior and doping use [11]. The Swiss program Cool & Clean [12] also emphasizes the promotion of life skills and personal responsibility among young people. The special element of this program is its inclusion of recreational drugs. The program integrates modules into different life settings such as schools, clubs and sports facilities [12]. Further programs in Iran [13] and Sweden [14], found different positive effects in amateur or hobby athletes. All of these (partially centralized) program ideas [15] could not, however, be transferred into the German sport organization and the federal school structures because of the large number of sport organizations and the decentralized approach to school curricula, which vary from state to state.

## Context of the research

Amateur and professional sports in Germany are regulated by 170 different organizations, which are all involved in doping prevention. In 2008, this decentralized prevention work was evaluated [16] with more than 1000 professional athletes, elite sport school students, journalists, medical care providers, stakeholders and trainers of these institutions, who were asked about the quality and quantity of the doping prevention activities and doping supporting structures [5, 17]. The results of this survey [18], led to the implementation of the National Doping Prevention Plan (NDPP) in 2009, with the aim of providing a new structure and system for doping prevention and education in the German professional and amateur sport systems. The changes proposed in the NDPP could be divided into two separate areas: structural changes in doping prevention and the development of prevention activities specifically targeted at young athletes.

### Structural changes due to the NDPP

First, doping prevention was centralized within 16 selected organizations (e.g., National Anti-Doping-Agency NADA, German Olympic Sports Federation DOSB, Federal Ministry of Interior BMI, Federal Health Ministry, etc.) in order for the NDPP to effectively reach the various target groups, and to help the plan receive financial grants to support prevention activities and measures [19]. Second, new rules and requirements for athletes and other stakeholders were implemented. For example: athletes received an athlete ID; athletes were obligated to follow more stringent doping control rules; sports federations and trainers could assign an ethical code; and other stakeholders were invited to participate in anti-doping campaigns. Third, within elite sports schools as well as in university education of sport scientists, the doping prevention time slots were expanded in the curriculum; anti-doping textbooks and teaching material were revised; and financial support was made available for anti-doping activities such as conferences.

### School-based prevention activities within the NDPP

Besides these structural changes for better coordination and reach, another important aim of the NDPP is to protect young people from substance use via education and the integration of new high-quality (understandable, age- and gender-specific, interactive) anti-doping activities and materials via training sessions, information sessions, and printed/online materials ([20], p.8). Emphasis is given to experiential learning, where participants learn via role-playing situations and the discussion of the resulting emotions [21]. As part of this objective, the National Anti-Doping Agency (NADA) - responsible for elite sports schools within the NDPP - developed two school anti-doping activities on the basis of the NDPP’s requirements: the “School Seminar” and the “Information Tour”. Both measures aimed to develop knowledge, critical awareness, and assertiveness, and to strengthen the young athletes’ character to prevent doping. The Information Tour included a presentation from an anti-doping official, a personal narrative from an elite athlete, and a doping control film. In addition, an information booth provided further teaching material to students. In the School Seminars students participated in full-day seminars on various topics related to doping. These seminars included students’ own presentations and role-playing-games. The information material that was developed for the school prevention programs included DVD, E-Books, websites, films, and paper brochures. The material was developed by the NADA in cooperation with experts from universities, the German Youth Sports Federation (DSJ) and the German Olympic Sports Federation (DOSB). Some material from the World Anti-Doping-Agency (WADA) and other national Anti-Doping-Agencies were combined and translated (e.g., from Norway and Austria, as well as Swiss E-Learning Tools). Both anti-doping activities were offered in elite sports schools, which in Germany are the most important educational institutions for future professional athletes. There are 43 elite sport schools across Germany with approximately 11,500 students (see DOSB), which are predominantly boarding schools for young athletes aged between 13 and 18 years. In these schools the athletes follow a normal state school curriculum that is adjusted to the training and competition schedule of the individual student (often including private tutoring if necessary).

To analyze whether these structural changes based on the NDPP were successful, Study I examined the extent to which the introduction of the NDPP led to structural changes in doping-prevention activities in elite sports schools. Study II examined whether new school based anti-doping activities such as the NADA Information Tour and School Seminar, led to improved knowledge about doping in participating athletes.

## Studies

To be able to compare the effects of the program, schools participating in the NADA program had to be matched to a (control) elite sport school in the same federal state (due to the state-specific curriculum). Therefore, at baseline all 43 elite sport schools in Germany were approached and a total of *N* = 36 (response rate: 88 %) schools responded. At the follow-up measurement 4 years later, 14 schools were eligible for the comparison measurement (7 control, 7 NDPP-program schools). This reduction was based on the necessity for state matching. Of these 14 schools three did not get permission from the federal ministry to participate and one was no longer willing to take part. This resulted in a final sample of *N* = 10 schools, of which *N* = 6 had participated in the NDPP program in the past 2 years and *N* = 4 had not been involved in NDPP activities in the previous 2 years. For both studies ethics approval was obtained from the internal ethics commission of the University of Potsdam, as well as from the federal Ministry of Interior (Az: SP6- 42009/7#5). In addition, informed consent was obtained from the school administration, and for Study 2, from the parents.

### Study 1 (teacher survey)

#### Materials and methods

##### Design

A longitudinal design (t0 = baseline, t1 = follow-up 4 years after NDPP introduction) was used to survey school staff to assess structural changes in doping- prevention activities in the school curriculum [22–24]. For Study I up to three teachers per school from the school sample described above who covered doping in their teaching were asked to evaluate different aspects of anti-doping teaching. These answers were compared to the answers of teachers in elite sport schools who had answered the same questions 4 years earlier.

##### Participants

*N*=22 school staff (response rate: 55 %, 17 male, 5 female, age: *M* = 49.1 ± 7.9 years) were surveyed in 2011 (t1). The same number of teachers from the same schools (but not necessarily the same teachers) were questioned in 2008 (t0). No information about sociodemographic variables was obtained.

##### Instruments

A questionnaire was developed to survey the school staff on the anti-doping activities within their school at t0 and t1. The questionnaire included 32 items, in which respondents evaluated statements about the frequency, type and scope of the school-based doping education activities, the quality of the teaching materials and the cooperation between the school and the NADA either by ticking a box if it applied to them (e.g., ‘Which of the following domains does your doping prevention work relate to? Please check: Ethical decision making; knowledge about medications, doping substances and methods; knowledge about health consequences of doping, other’) or by rating the methods and materials used on a six point Likert-scale ranging from 1 (*Very good*) to 6 (*Very bad*) (E.g. Which sort of educational materials did you use in the 12^th^ class (age 17 years.)? Please name and rate it). The questionnaire can be found in Additional file 1. For further information see also Wippert et al., 2008 [16]. The questionnaires were sent to the school’s anti-doping-officials, at t0 and t1. They were instructed to fill out one questionnaire themselves and forward two questionnaires to two other teachers in their school that were involved with anti-doping activities at t0 and t1 along with stamped return envelopes. All questionnaires were completed anonymously.

##### Statistics

All data were analyzed descriptively (mean values, standard deviations) in SPSS 22. To examine changes in the anti-doping activities in the schools at t0 and t1, McNemar tests were performed for binary variables. Due to the non-normal distribution of data, Wilcoxon tests were performed for the scale-based responses.

#### Results

Results of the school teacher survey are reported in Table 1. 82 % of school teachers reported that the topic of anti-doping was present in the curriculum after the introduction/start of the NDPP (t1), whereas 64 % reported integration of anti-doping activities in the school curriculum at t0 (McNemar-Test, *n.s.*). Instruction time at t0 on average amounted to *M* = 2.71 ± 2.41 h and *M* = 2.56 ± 4.25 h at t1 (Wilcoxon-Test, *Z* = −1.304; *n.s.*). The anti-doping efforts at both t0 and t1 were reported to be mainly directed towards students, rather than teachers (t0: in 40 % of schools, t1 in 27 % of schools; McNemar-Test, *n.s.*) and parents (t0 and t1 both at 33 % of schools). The content of the schools’ anti-doping efforts concerned primarily the ethical aspects of doping (t0: 93 %, t1: 94 %; McNemar-Test, *n.s.*), health consequences (t0: 93 %, t1: 81 %, McNemar-Test, *p* = *n.s.*) and providing students with additional information on pharmaceuticals, medications and methods (t0: 86 %, t1: 81 %, McNemar-Test, *p* = *n.s*.). There was no significant difference observed.

**Table 1:** Comparison of anti-doping prevention work in schools between 2008 and 2011 (*N* = 22)

Question in Questionnaire	Yes answers 2008 in %	Yes answers in 2011 in %	McNemar Test on difference
Is the anti-doping subject incorporated in the school’s curriculum?	64	82	0.38
Doping activities in school are directed toward students	40	27	1.0
Doping activities in school are directed toward parents	33	33	1.0
Educational anti-doping work in school is directed toward imparting knowledge about ethical aspects of doping	93	94	1.0
Educational anti-doping work in school is directed toward imparting knowledge about health consequences of doping	93	81	0.25
Educational anti-doping work in school is directed toward imparting knowledge about pharmaceuticals, medications and methods	86	81	1.0
Does the school use new developed teaching documents for doping prevention work in grade 11?	41	79	0.06
Does the school use web based educational material for doping prevention work in grade 11?	63	21	0.13

The school officials were also surveyed about their evaluation of the prevention materials. Teachers reported a trend in the increase of the usage of newly developed teaching materials 4 years after NDPP introduction in grade 11 (age 16) (reported use at t0: 41 %; at t1: 79 %, McNemar-Test*, p =* 0.06). The material provided for doping prevention work, such as teaching documentation, books or informational material (in the form of magazines or brochures) was rated in general to be more effective at t1 than at t0. But there was no significant difference as well as no significant decrease in the usage of web-based educational work in grade 11 (reported use at t0: 63 %; t1: 21 %; McNemar-Test, *n.s.*).

#### Summary

The results of the survey among teachers engaged in doping prevention work in elite sport schools indicate that the introduction of the NDPP did not lead to significant curricular changes in doping prevention activities in elite sport schools. Only a trend of increased usage of the recommended teaching materials was observed.

### Study II (student survey)

#### Materials and methods

##### Design

A cross-sectional control-group design was used to evaluate the effects of the NADA Information Tour and School Seminar on students (within the same schools and same time of t1 of study I). Students had either participated in at least one NADA activity (NADA information tour and/or NADA School Seminar) in the past 2 years (NADA group) or had not participated in any of the NADA activities (comparison group). The groups were grade-matched. The NADA measures were launched in the first 2 years after the NDPP introduction, thus students had participated around 2 years before the survey. Both NADA measures were planned and organized by NADA personnel and took place in classrooms at the schools. The materials were also developed by NADA personnel and brought along to the sessions (see Introduction for more information on the content of the NADA activities). For Study II, students from 10 schools (see school sample description above) were surveyed at t1, and the results of the students that had participated in additional NADA anti-doping activities (School Seminar or Information Tour) were compared to students that had not participated in any of these activities.

##### Participants

Initially we planned to include 300 students (allowing us to observe middle strong effects (*r* > 0.3) with a power of greater than 0.8), half of them having participated in at least one NADA project activity. To do so we asked the contact person in every school to forward the student questionnaires to thirty students (preferably in grade 10 and 11, so students would be halfway through their school attendance). For the NADA schools, the students had to have taken part in at least one of the NADA activities. In total, 220 students returned the questionnaire, but seven students had to be excluded because they did not specify whether they had participated in the anti-doping activities. This led to a final inclusion of *N* = 213 students (response rate 65.5 %, completion rate 98.6 %, 54.5 % male, 45.5 % female, age *M* = 16.7 ± 1.3 years, grades 8–13, Comparison group *N* = 111, NADA group *N* = 102). In the NADA-group 52 students participated in the “Information tour”, 20 in the “School seminar” and 30 in both projects. Since we wanted to focus on the general effect of the NADA projects, we did not distinguish between the participation in either of these activities. The NADA group and the comparison group were comparable in terms of school grade (NADA group *M* = 11.1, comparison group *M* = 11.3), age (NADA group *M* = 16.6, comparison group *M* = 16.8) or sex (NADA group 55.9 % male, comparison group 53.2 %), but the NADA group included more competitive athletes (NADA group: 95.1 %, comparison group: 86.5 %; *p* = 0.03).

##### Instruments

A questionnaire was constructed to survey students which assessed their level of knowledge on the topic of doping along with a subjective assessment of NADA prevention measures including quality, emotional involvement and the development of critical awareness or assertiveness. The knowledge test consisted of nine multiple-choice questions relating to various aspects of doping. The questions were designed on the basis of content analyses of the prevention materials that were used in the NADA prevention courses (Information Tour and School Seminar). The questions increased in difficulty within various doping topics (e.g., rules, substance groups, mechanisms). There were three available answers for each multiple-choice question, one of which was correct. An example being: “*Which of the following drugs or ingredients are forbidden by the WADA code*? *ACC akut or Buscopan or Clenbutero*l?” In addition, two open questions asked participants to list alternatives to doping and sources of information about anti-doping. The questionnaire can be found in Additional file 1. For the student-knowledge questionnaire a total sum score was calculated for the number of correct answers. Questions that were not answered were marked as wrong. This resulted in a score between 0 and 11 points for every student; higher scores indicated more knowledge about doping.

##### Statistics

All data were initially analyzed descriptively (mean values, standard deviations) in SPSS 22. An Analysis of Covariance (ANCOVA) was performed in order to assess whether students who had participated in NADA activities had significantly more knowledge about doping than those who did not. To determine the degree to which the differences were influenced by interacting variables, ANCOVA was performed using age, sex and competition level as covariates (with the latter being operationalized as the dichotomous question: “are you a competitive athlete?”).

#### Results

To assess if students who participated in at least one NADA measure had better knowledge about doping we performed an Analysis of Covariance (ANCOVA) using age, sex and competitive competition level as covariates. This leads to an adjusted mean of 6.51 ([95 % CI 6.25 to 6.77]) on the knowledge test for the comparison-group compared to 7.64 ([95 % CI 7.36 to 7.91]) for the participants of NADA group. This difference is statistically significant (*F(1, 207) =* 33.99, *p* <0.001), meaning that participants in the NADA group on average scored better in the knowledge test than participants in the Comparison group, even when we controlled for the abovementioned variables.

#### Summary

The findings of a survey among 213 elite sport school students indicated that students that had participated in at least one of the two NADA anti-doping activities (seminar or tour) scored higher on a doping-knowledge test than students who had not participated in any of these NADA events.

## General discussion

An evaluation among elite sport schools 4 years after the introduction of the NDPP showed slight but not significant structural changes in frequency and duration of doping prevention activities in elite sport schools. However, an improvement in knowledge communication due to the new instructional materials was observed. Although was only a trend, the revised teaching and informational materials were better understood, received better evaluations and were considered more appropriate for the target group, which are an important first steps toward for effective doping prevention [25, 26]. A better understanding and knowledge enable athletes to make an informed decision on the properties and risks of doping issues [27, 28], yet information about doping is often not presented in an easy to understand and engaging form [29]. Thus, with regard to the first study objective, it can be concluded that the NDPP policy to target young athletes during a period in their lives when attitudes and values are forming, supported a higher quality of education [30], but failed to lead to structural effects in schools (e.g., prevention duration, trained staff quality).

Study II showed that students who participated in newly designed additional NADA school anti-doping activities (“Information Tour” and “School Seminar”) had a slight but significant higher level of knowledge about doping than students who had not participated, although both groups used the new and improved material of the NDPP in the everyday prevention work in school as described above. The additional two NADA activities focused on interactive and participatory forms of dealing with role-playing and emotions [21] and may explain the significantly better performance on the knowledge test among students who participated in those campaigns compared to students who did not [30]. Moreover, the knowledge test results were obtained up to 2 years after participation in the anti-doping programs, suggesting that the effect has a satisfactory retention. There are several explanations: first recent studies show, that providing interactive material [30] or universal social and emotional learning programs [31] support the improvement of social and emotional skills, attitudes and behavior – which may be longer lasting than mere knowledge of rules and regulations and that were also captured by the knowledge test [25, 26]. Second, prevention programs that are related to specific (school) settings show stronger effects in comparison to general population or universal prevention programs. This effect was also found with the U.S. doping prevention programs ATLAS and ATHENA [10] and has been documented for other types of substance abuse prevention programs (e.g., tobacco, [32]). Thirdly, incorporating “booster sessions” to reinforce key messages is suggested for doping prevention [30] and could be an explanation for the better performance of athletes who participated in the additional NADA anti-doping activities in addition to the regular anti-doping curriculum offered at their school.

Although results appear positive at first, a discussion concerning the cost-benefit relationship of such national prevention guidelines is certainly required. The introduction of national guidelines is an extremely difficult structural endeavor in a federally organized and thus de-centralized school system, as is clear from the low level of structural change that was observed. Another point of consideration is the cost of the additional NADA campaigns such as travel costs to the several schools throughout Germany and the respective materials. The prime challenge in implementing good prevention work remains the structural weaknesses of the decentralized German system of elite athlete development, which operates in near-isolation from the school system. In this context, the results of doping prevention on the basis of the NDPP can be viewed as a positive.

Methodologically, there are some limitations. In the first study there are a) confounding factors because of differences between schools and locations cannot be ruled out. Further b) large standard deviations limit the interpretation/conclusions about structural changes. Due to the c) cross-sectional design in the second study a causal conclusion about the improvement or program effects is not possible. Further d) no standardized knowledge tests or standardized testing procedures on the topic of doping are available [25, 26]. These limit the interpretation and comparison of the results with other programs. Finally, e) This survey only assessed knowledge and attitudes, which do not necessarily translate into action and actual decreased doping use.

## Conclusion

Within a 4-year follow-up frame the present study gives an impression of how a national guideline (and a national restructuring process) can influence frequency, type, scope and quality of anti-doping prevention work within an elite sports school setting. The first study investigated whether the restructuring of the national anti-doping prevention would lead to an improvement of doping prevention activities in elite sport schools; the second study investigated whether the new additional NADA anti-doping activities would improve knowledge about doping and health among young athletes in elite sport schools. In total 213 students and 22 school teachers doing doping prevention work gave feedback about their objective and subjective impressions. Our main results are the findings that, despite the enormous national effort, there were only limited structural changes in the frequency, type, and scope of doping prevention in elite sport schools are limited. However, the prevention materials developed within the NDPP were rated higher-quality and more frequently used in the schools. Finally, students who participated in additional interactive NADA anti-doping activities (booster sessions [30]) showed a higher knowledge about the topic of doping and health, which is valuable in a prevention context [25–28].

It also became clear that the degree to which prevention work is centralized could be an important factor in the success of anti-doping initiatives in any country. It should be noted that the most effective doping prevention is likely to take place at different structural levels, which can take an extremely long time. Likewise, its focus cannot be restricted to attitude or to any single measure or program. Further research is necessary to understand the interaction between the different prevention levels with respect to the social structures of specific settings or countries.

## Additional file

Additional file 1: Knowledge test (section A of the questionnaire). (DOC 47 kb)
