# Supplementary material for: National doping prevention guidelines: Intent, efficacy and lessons learned - A 4-year evaluation
Source: Subst Abuse Treat Prev Policy. 2016 Oct 10;11:35. doi: 10.1186/s13011-016-0079-9 (PMC5057456; doi:10.1186/s13011-016-0079-9)
Supplement: Additional file 1: — Knowledge test (section A of the questionnaire). (DOC 47 kb) [file 13011_2016_79_MOESM1_ESM.doc]

Knowledge test (section A of the questionnaire)

1. Wissen über Doping

Kreuze bitte jeweils die richtige Antwort an (Hinweis: Es ist immer nur eine Antwort richtig).

| 1. Wenn ein Sportler gar nicht weiß, dass er durch das Passivrauchen von Cannabis, oder den Verzehr von Mohnkuchen positiv getestet werden kann, dann | | |
| --- | --- | --- |
| kann er bei einem positiven Ergebnis trotzdem bestraft werden | kann er nicht bestraft werden | wird er auf jeden Fall freigesprochen |
| 1. Ein besonders durch den Radsport bekannt gewordenes Dopingmittel ist | | |
| LSD | EPO | THC |
| 1. Wie gehen Kontrolleure bei einer Dopingkontrolle vor? | | |
| Sie sprechen den Kontrolltermin vorher mit dem Trainer ab | Sie melden sich 24 Stunden vor der Kontrolle telefonisch | Sie besuchen den Sportler ohne Voranmeldung zu jeder Zeit |
| 1. Drogen wie Amphetamine, Kokain, Ecstasy, aber auch Arzneistoffe wie Ephedrin, gehören zu der Substanzgruppe der | | |
| Anabolika | Stimulanzien | Diuretika |
| 1. EPO ist ein Hormon, das in der Niere gebildet wird und die Aufgabe hat, … | | |
| die Produktion der weißen Blutkörperchen (Leukozyten) anzuregen | die Produktion der roten Blutkörperchen (Erythrozythen) zu steigern | die Produktion der Blutplättchen (Thrombozyten) zu regulieren |
| 1. Welches Medikament, bzw. welcher Inhaltsstoff steht auf der Verbotsliste? | | |
| ACC akut | Buscopan | Clenbuterol |
| 1. Welche Alternativen zu Doping fallen Dir ein? | | |
|  | | |
| 1. Welche Informationsquellen zum Thema Anti-Doping kennst Du? | | |
|  | | |
| 1. Wessen Pflicht ist es, sich jeweils aktuell darüber zu informieren, welche Substanzen verboten sind und worin sie enthalten sind? | | |
| Die meines Trainers. Er muss diese Informationen an mich weitergeben | Es ist meine eigene Pflicht | Die des Doping-Kontrolleurs. Er klärt mich bei jedem Besuch über die aktuelle Verbotsliste auf |
